# Biomechanical characteristics of tibio-femoral joint after partial medial meniscectomy in different flexion angles: a finite element analysis

**DOI:** 10.1186/s12891-021-04187-8

**Published:** 2021-04-02

**Authors:** Xiaohui Zhang, Shuo Yuan, Jun Wang, Bagen Liao, De Liang

**Affiliations:** 1 Guangzhou University of Chinese Medicine, 12 Airport Road, Guangzhou, 510405 Guangdong Province China; 2 Department of Sports Medicine, Guangzhou Sport University, 1268 Guangzhou Avenue 1268, Guangzhou, 515500 Guangdong Province China; 3 Gaosun Medical Imaging Diagnosis Center of Guangdong Province, 117 Liuhua Road, Guangzhou, 515500 Guangdong Province China

## Abstract

**Background:**

Recent studies have pointed out that arthroscopy, the commonly-used surgical procedure for meniscal tears, may lead to an elevated risk of knee osteoarthritis (KOA). The biomechanical factors of KOA can be clarified by the biomechanical analysis after arthroscopic partial meniscectomy (APM). This study aimed to elucidate the cartilage stress and meniscus displacement of the tibiofemoral joint under flexion and rotation loads after APM.

**Methods:**

A detailed finite element model of the knee bone, cartilage, meniscus, and major ligaments was established by combining computed tomography and magnetic resonance images. Vertical load and front load were applied to simulate different knee buckling angles. At the same time, by simulating flexion of different degrees and internal and external rotations, the stresses on tibiofemoral articular cartilage and meniscus displacement were evaluated.

**Results:**

Generally, the contact stress on both the femoral tibial articular cartilage and the meniscus increased with the increased flexion degree. Moreover, the maximum stress on the tibial plateau gradually moved backward. The maximum position shift value of the lateral meniscus was larger than that of the medial meniscus.

**Conclusion:**

Our finite element model provides a realistic three-dimensional model to evaluate the influence of different joint range of motion and rotating tibiofemoral joint stress distribution. The decreased displacement of the medial meniscus may explain the higher pressure on the knee components. These characteristics of the medial tibiofemoral joint indicate the potential biomechanical risk of knee degeneration.

## Introduction

Arthroscopic partial meniscectomy (APM), a most commonly-performed orthopedic surgery, is often adopted to treat traumatic meniscus tears that usually occur in physically active individuals. Recently, several high-quality randomized controlled trials (RCTs) challenge the indications of APM [1, 2], and there is growing concern about APM because patients treated with APM may face an increased risk of developing knee osteoarthritis (KOA).

Biomechanical factors may contribute to the initiation and progression of KOA. Though the evidence supporting the relationship between long-term physical activity and structural KOA progression in patients with knee pain is scarce [3], there is still concern that physical activity may aggravate cartilaginous damage in patients after APM. Elucidating the stress characteristics of articular cartilage can reveal the biomechanical nature of KOA. In-depth understanding of stress transfer in the articular cavity after APM may illuminate the biomechanical causes of OA progression [4, 5].

Finite element method (FEM) can incorporate complex anatomical shapes, load scenes, boundary conditions and bone characteristics [6]. These parameters can be examined for their influence on the expected results [7–12]. Simulation using finite element (FE) models can provide intuitive graphical results to explain the biomechanics changes after APM [13].

Few studies have explored the stress on knee joints with different range of motion after APM. The construction of finite element analysis models with different joint ranges of motion could illustrate the influence of load on knee cartilage stress under different conditions. We hypothesized that the overload of the knee was related to the contact area of the cartilage as the range of motion of the joint changes. The elucidation of this mechanical feature might help to understand the biomechanical process of KOA. Therefore, the purpose of this research was to construct a detailed 3D FE model of the knee after APM, including bones, major ligaments, cartilage, and meniscus. Then the characteristics of the load on the tibiofemoral articular cartilage and meniscus displacement in different flexion and rotation ranges were analyzed using the model.

## Methods

### Data acquisition

All methods were carried out in accordance with the Declaration of Helsinki.

The magnetic resonance data were obtained from a 35-year-old male (body weight 60 kg, height 172 cm) who had received partial medial meniscectomy (PMM) using an MRI scanner (SIEMENS 3.0 T Skyra, Germany). During the scan, the subject was in a lying position, and 3D proton density-weighted imaging sequence was selected. A dual-source 128-slice CT equipment (SIEMENS Definition Flash CT, Germany) was used for computed tomography scan of the same subject. The lower extremities were scanned in a neutral posture with a slice distance of 0.625 mm and a field of view of 500 mm.

### 3D reconstruction of the knee

MIMICS 19.0 (Materialise, Leuven, Belgium) was used to reconstruct 3D models of bone structure and soft tissues. The DICOM image files were imported and segmented according to the gray intensity. Then, the computer tomography bone segmentation program was used to perform a separate 3D reconstruction of each bone. Next, the MRI images of the articular cartilage (femur, tibia, and patella), meniscus (medial and lateral) and ligaments (medial collateral, lateral collateral, anterior cruciate, posterior cruciate and patellar tendon) were segmented. In order to minimize the deviation in the model, experienced orthopedic physicians and radiologists manually segmented the skeletal and non-skeletal structures with an accuracy of 0.1 mm. Finally, a model of the knee joint after PMM was constructed. The medial meniscus was cut out by 2/3, leaving only the edges near the joint capsule.

### FE modelling and material properties

The MRI images were imported into Mimics 20.0 software in DICOM format, and the 3D model was synthesized from the layers. Then, the model was smoothed, amended and spherized using Geomagic Studio (version 2015, Geomagic, SC, U.S.A.). The solid model of cortical bone, cancellous bone, articular cartilage, and meniscus was generated by Solidworks CAD software (version 2017, SolidWorks Corp, Dassault Systèmes, Concord, MA). The geometric model was imported into ANSYS 17.0 finite element analysis software to establish the analysis model, and the material property parameters of bone, meniscus, articular cartilage, ligament were imported into the material library for analysis.

Since this study focused on the stress and relative movement of articular cartilage and meniscus, the bone deformation was not taken into consideration. The bone part was regarded as compact and assumed to be homogeneous and isotropic. Because obvious changes would not occur in viscoelastic material after a short-term loading, the articular cartilage and meniscus were defined as linear elastic and isotropic materials and ligaments were regarded as super-elastic and isotropic [14–16] The material properties as described in the previous literature were specified in Table 1.

**Table 1:** Material properties of bones, cartilage, and ligaments

	Element type	Youn’s modulus (MPa)	Poisso’s ratio	Node number	Elements
bones	Solid185	17,000	0.30	9771	30,647
Meniscus	Solid185	59.0	0.49	1683	4703
cartilage	Solid185	5.0	0.46	6515	18,617
ligament	Solid185	215.3	0.40	7536	21,678

### Loads and boundary conditions

The boundary conditions of the three-dimensional finite element model were as follows: The femur was unconstrained, and the tibia was to buckling degrees of freedom and three translational degrees of freedom constraints [14, 16–18]^.^

**Fig. 1:**
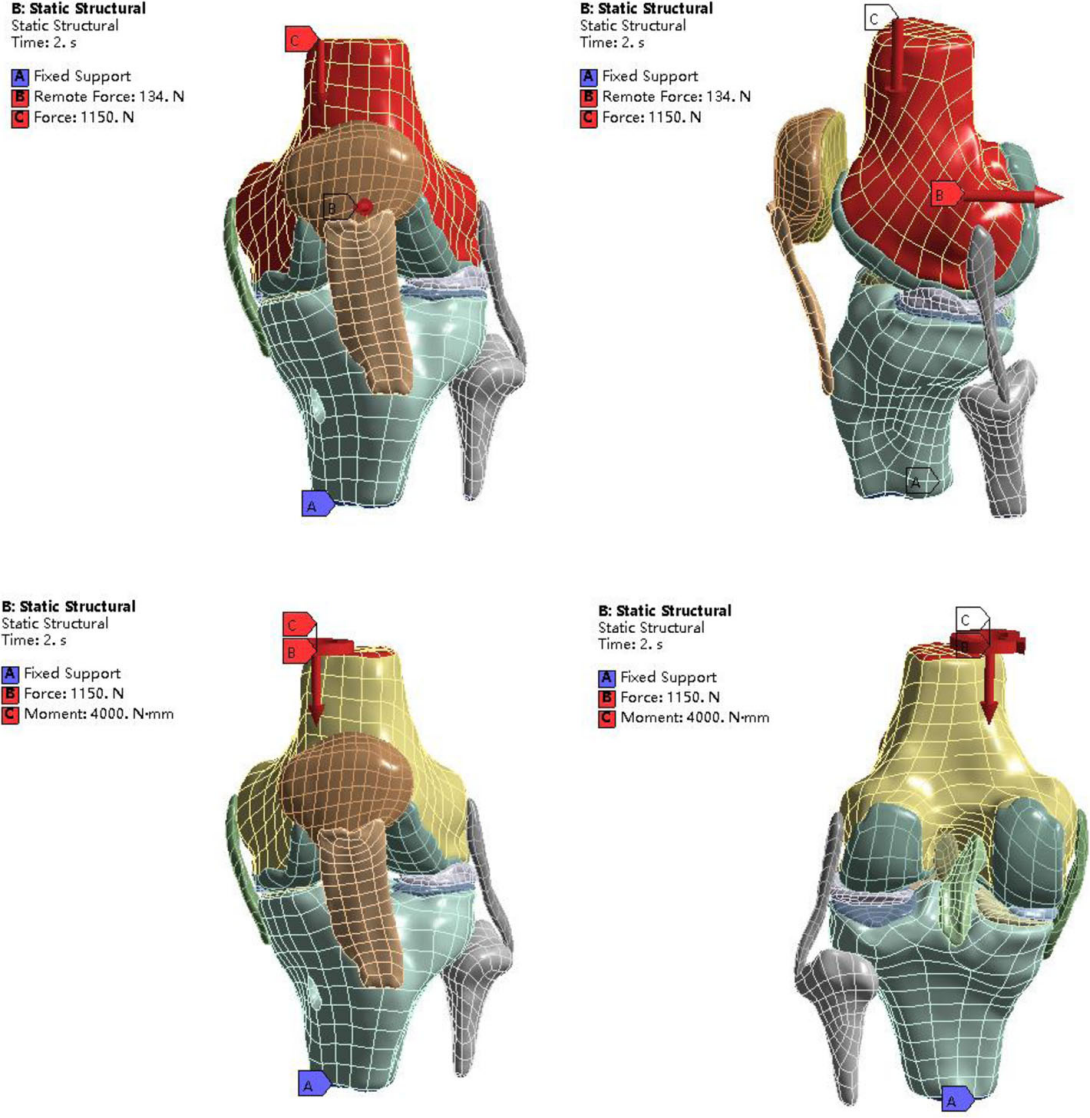
The view of 3D models used in the FE simulation

The distal bone and fibula were fixed, along the line connecting the inner and outer condyles of the femur to perform 0°, 30°, 60°, 90° rotation, imitating the knee flexion neutral position to apply load. The value of the force applied was based on the research of Ahmed [14] and Kutzner [19]. A load was applied on the top section of the femur in the three-dimensional finite element model of the knee joint. A compressive force of 1150 N (two body weights) was applied in the vertical downward direction. (1) A 134 N femur posterior thrust was applied and loaded at the midpoint of the line connecting the midpoints of the inner and outer condyles of the femur, along the direction perpendicular to the coronal plane. (2) Internal-external torque of 4 Nm was compressed to simulate the internal and external rotation of the knee joint (Fig. 1).

### Analysis

The stress (von Mises stress) of tibiofemoral articular cartilage and the displacement of meniscus were observed during the simulation of knee motion and rotation after APM.

## Results

### Validation of the three-dimensional finite element model of the knee

The model in this experiment was basically the same as the finite element model by Shirazi with the same boundary conditions and neutral position 0 [20]. But the maximum stress was greater than the normal knee model by Wang [21]. Therefore, the effectiveness of our model could be assessed by comparison.

### Establishment of three-dimensional finite element model of knee joint

The total number of elements was 582,044 and the total number of nodes were 391,670. This constructed knee joint entity was a three-dimensional finite element model that highly simulated the structure and material properties of the knee joint.

### Maximum contact stress of the tibiofemoral articular cartilage and meniscus

The maximum stress of the cartilage on the inside of the tibiofemoral joint was higher than that on the outside, except when flexed and loaded with external rotation.

**Fig. 2:**
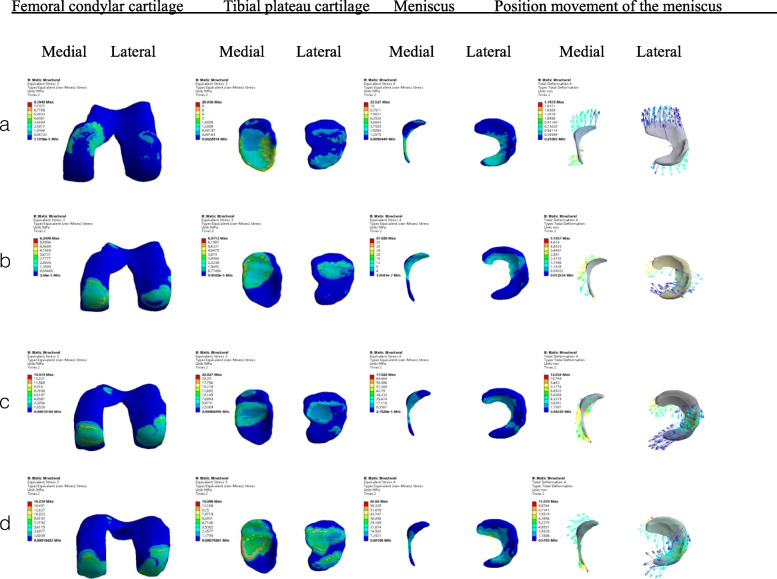
Results of contact stress of tibiofemoral articular cartilage and meniscus displacement under a combined load of 1150 N in compression, 134 N posterior on the femur. Figures **a**-**d** are the stress cloud diagrams of the tibiofemoral joint at 0°, 30°, 60°, and 90° knee flexion. The colour changes (from red to deep blue) represent the stress variation (from large to small) on the stress nephogram

Generally, the maximum stress increased with the increased degree of flexion. What caused concern was when the knee was flexed at 0°, during which the maximum stress value of the medial tibial plateau cartilage was 4.3–4.8 times that of the lateral. At the same time, the maximum stress value of the medial femoral condyle cartilage at 0° flexion and external rotation was more than 8 times that of the outer side, while at 0° flexion and internal rotation, the stress of the medial side was only 0.65 times that of the outer side (Fig. 2; Tables 2, 3, 4).

**Table 2:** The effect of the combined complication of 1150 N compression and posterior 134 N on maximum contact stress in knee

Flexion angle	FC		TC		Meniscus	
	Medial	Lateral	Medial	Lateral	Medial	Lateral
0°	8.7049	1.771	20.936	2.4156	13.904	23.521
30°	6.2498	3.6311	6.9713	4.8119	34.298	97.928
60°	14.874	11.865	22.827	14.006	77.022	33.809
90°	14.887	13.923	16.086	12.489	36.116	65.62

*FC* femoral cartilage, *TC* tibial cartilage

**Table 3:** The effect of the combined complication of 1150 N compression and 4 Nm internal rotation torque on maximum contact stress in knee

Flexion angle	FC		TC		Meniscus	
	Medial	Lateral	Medial	Lateral	Medial	Lateral
0°	8.3981	1.9586	19.092	2.3646	12.734	23.919
30°	6.1423	3.0192	7.3016	4.4836	34.116	95.843
60°	14.822	11.386	22.957	13.384	80.894	33.143
90°	14.776	13.427	16.747	11.985	36.16	65.266

*FC* femoral cartilage, *TC* tibial cartilage

**Table 4:** The effect of the combined complication of 1150 N compression and 4 Nm external rotation torque on maximum contact stress in knee

Flexion angle	FC		TC		Meniscus	
	Medial	Lateral	Medial	Lateral	Medial	Lateral
0°	6.6193	1.402	4.6624	7.1584	10.315	1.9374
30°	5.9945	4.0824	34.494	98.511	6.3055	4.7154
60°	14.137	11.431	73.479	31.241	22.26	13.937
90°	18.231	15.827	31.381	53.557	15.478	17.358

*FC* femoral cartilage, *TC* tibial cartilage

In this model, the maximum stress of the meniscus peaked at 30°flexion of the knee, when the lateral meniscus reached 97.92 kpa. The maximum contact stress of the lateral meniscus was greater than that of the medial, except when the knee was at 60° flexion, and the opposite was true. The maximum contact stress of the lateral meniscus was 2.8–2.9 times that of the medial when the knee was flexed at 60° and internally rotated. On the contrary, when flexion is 60°, the maximum contact stress of the medial meniscus was 2.4 times larger than the lateral. The maximum stress was concentrated at the margin where the medial meniscus had been excised (Fig. 2; Tables 2, 3, 4).

### Meniscus displacement (mm)

When the knee joint was flexed and rotated at the same time, the displacement of the lateral meniscus was greater than that of the medial. The lateral meniscus had the largest displacement at 60° knee flexion, followed by 90°, and the smallest displacement at 0°.

**Fig. 3:**
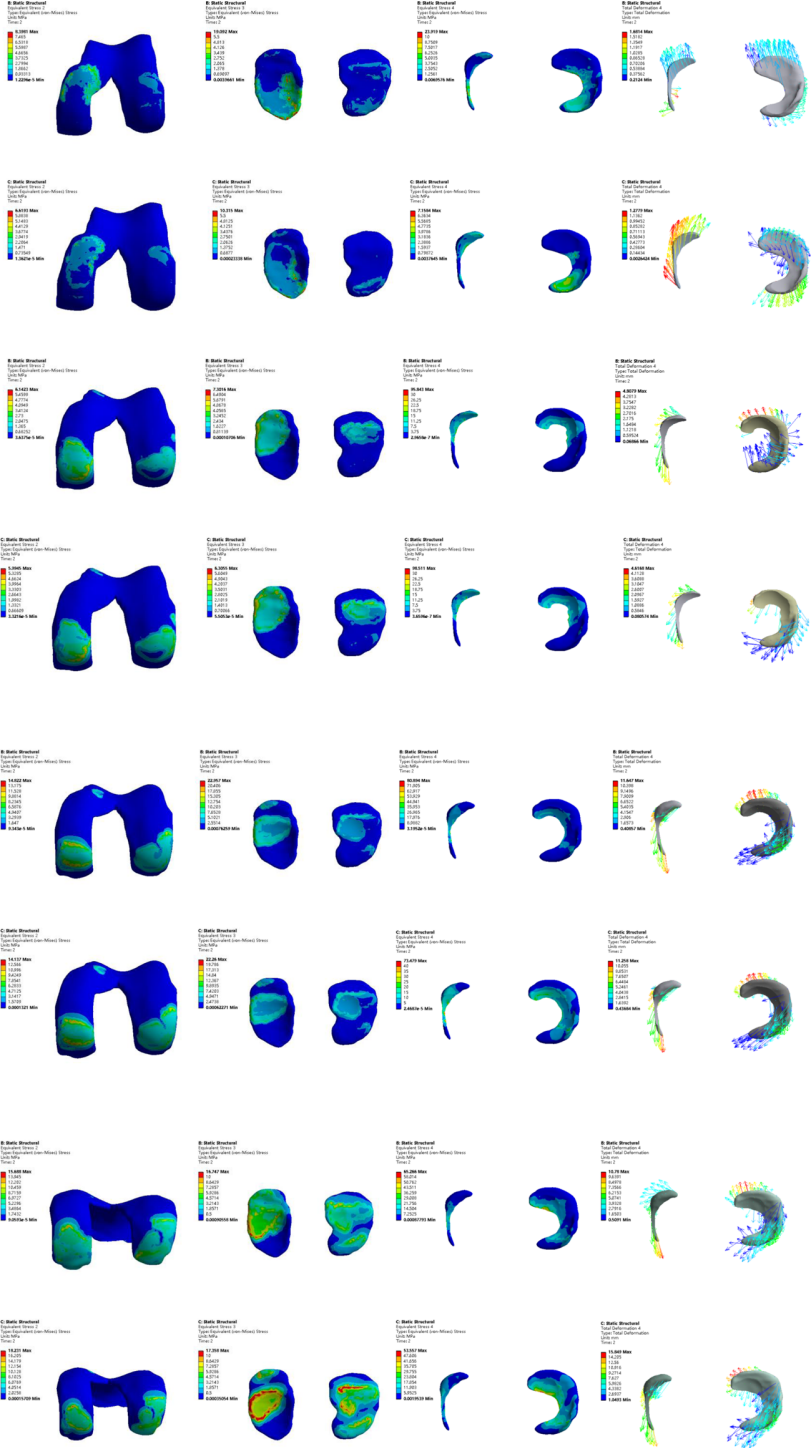
Results of contact stress of tibiofemoral articular cartilage and meniscus displacement under a combined load of 1150 N in compression, 134 N posterior on the femur and an internal torque of 4 Nm. **a**, **c**, **e** and **g** indicate knee flexion at 0°, 30°,60°, and 90° with external rotation, respectively. **b**, **d**, **f** and **h** respectively indicate the above-mentioned conditions at different degrees of knee flexion and internal rotation. The colour changes (from red to deep blue) represent the stress variation (from large to small) on the stress nephogram

When the knee joint was flexed and rotated at the same time, the lateral meniscus displacement was also greater than that of the medial. The displacement of the meniscus increased with the increase of the flexion angle. When the knee was flexed at 90°, in external and internal rotation, the maximum displacement of the lateral meniscus was 15.85 mm (Fig. 2and 3).

## Discussion

A long-term follow-up study of the clinical and radiological outcomes of arthroscopic meniscectomy indicated that the type of meniscus lesion, older age, or intraoperatively described significant cartilage damage were not the most radiologically critical factors for the development of osteoarthritis [22]. It was implied that biomechanical factors might trigger the development of KOA. In this study, a high-fidelity three-dimensional finite element model of the knee joint (including bone, articular cartilage, meniscus, and major ligaments) after PMM was developed. The main purpose was to compare the effects of the different flexion angles, as well as internal and external rotations on the contact stress of the tibiofemoral joint.

The present study demonstrated that the maximum stress of medial plateau cartilage was higher than that of the lateral, and it increased with the increased angle of knee flexion. However, this characteristic did not apply to the meniscus and femoral cartilage. Previous studies have shown that the medial meniscus is more important than the lateral, for it restrains uniplanar anterior loads on the tibia [23]. Furthermore, compared with the lateral meniscus resection, the biomechanical changes of the medial meniscus resection are more significant, which is why we have chosen medial meniscectomy as the target of this study. Our study confirmed the hypothesis that tibiofemoral articular cartilage contact area overload was related to knee range of motion.

Under a 1150 N compression combined with 4 Nm internal rotation load, the maximum stress of the lateral meniscus was greater than the medial at 0°, 30°, 90° flexion. At 60° flexion, the maximum stress of the medial meniscus was greater than the lateral. For femoral condyle and tibial plateau at different knee flexion angles and rotation, the maximum stress on the inside of the cartilage was greater than that on the outside. In case of 1150 N load combined with 4 Nm external rotation, the maximum stress on the outside of the tibial plateau was greater than the inside when the knee was flexed at 30° and 90°. The maximum stress on the tibial plateau cartilage at 0° and 60° was greater on the inside than the outside, and at 0°, 30°, 60° and 90°, the maximum stress on femoral condyle cartilage and the meniscus was greater on the inside than the outside. The finite element simulation results of the model straightening at 0° were similar to the results of previous studies on peak stress. But with the increase of the flexion angle, the maximum stress of articular cartilage and meniscus in our model was greater than the healthy one [16, 21]. Moreover, the maximum stress gradually moved backward as the angle of flexion increased. Our results indicated that more stress was concentrated on the edge of the removed meniscus and the maximum stress of the medial tibial plateau increased, which could explain the mechanical mechanism underlying the progression of KOA.

The maximum stress value of the medial tibial plateau cartilage was 4.3–4.8 times that of the lateral when the knee was flexed at 0°. At the same time, the maximum stress of the medial femoral condyle cartilage at 0° flexion and external rotation was more than 8 times that of the outer side, except for 0° flexion and internal rotation, while the maximum stress of the medial side was only 0.65 times that of the outer side. When combining internal and external rotation under different joint flexion degrees, in most cases, the maximum stress on the medial cartilage of the knee joint was greater than that on the lateral side. The above results showed that the significant increased stress on medial components (including cartilage and meniscus) was caused by the teared medial meniscus, which was consistent with the previous studies [13, 24]. The stress concentration directly indicated that the abnormal overload could damage the risky area.

When vertical and forward loads are applied to the knee, the intact meniscus exhibits compression and displacement to provide adequate contact area between the cartilage of the femoral condyle and the tibial plateau. The meniscus bears stress, absorbs shock, and disperses stress by deformation. Previous studies have shown that the contact pressure of the normal knee joint medial compartment is greater than that of the lateral compartment in a healthy person, and the medial meniscus bears more mechanical effects [25, 26]. Consistent with the results in previous health models, our study showed that the maximum stress of the medial tibial plateau, including cartilage and meniscus, was greater than that of the lateral [21]. Generally, the current study demonstrated that the maximum stress on the lateral meniscus was greater than that on the medial side, except in a knee flexion of 60°. However, the opposite occurred in tibial plateau cartilage, demonstrating that the maximum stress was larger on the medial side. The reason could be that the circumferential bearing capacity of the medial meniscus was weakened after partial resection of the medial meniscus, and thus the effect of shock absorption and pressure was attenuated, showing that the maximum medial contact stress of tibiofemoral articular cartilage was greater than the outer side. Based on the results, it is suggested that evaluating the effect of load at different angles of joint motion on knee cartilage may help to identify the risk factors for the development of KOA, even if the knee is stable and functions well.

When the knee was flexed and rotated internally and externally, the maximum displacement of the lateral meniscus was greater than that of the medial side. The maximum displacement of the meniscus increased with the rise of the flexion angle. It was suggested that in knee joint flexion and rotation, the healthy side of the meniscus bore a larger load, which could reduce the stress load of tibiofemoral articular cartilage. Under different degrees of knee flexion and rotation, the removed medial meniscus only bore part of the load on the free edge. These results indicated an increase in the direct contact area of the medial tibiofemoral joint cartilage.

Previous studies have shown that without the shock absorption by the meniscus, increasing the direct contact area between the femoral condyle cartilage and the tibial plateau leads to elevated stress, which can result in early cartilage degradation and early-onset osteoarthritis [13]. Our results illustrated the compression of cartilage and meniscus after partial meniscus surgery in detail. As the degrees of flexion and rotation increased, more stress was concentrated on the medial tibial plateau and the edge of the meniscus. This increase in stress could lead to early proteolytic degradation of the meniscus matrix and articular cartilage, thereby reducing the tensile strength. Although the characteristics of cartilage stress and meniscus displacement in the present model only reflect the transient response of the knee joint under compression load induction, previous studies have suggested that higher shear stress may cause early proteolytic degradation of the meniscus matrix and the tension of the articular cartilage may reduce the strength [26]. The peak shear stresses on the meniscus showed an obvious increase, and that on the cartilage was slightly increased.

Early studies have supported APM as a standard procedure, although some patients may experience poor outcomes due to joint instability [27]. Yet, other clinical studies have shown that despite the well-known benefits and general acceptance of arthroscopic partial meniscectomy, the procedure cannot be considered benign considering the consequent development of osteoarthritis in patients [22, 28]. We believe that the relationship between knee flexion load and articular cartilage maximum stress as revealed in this model can be helpful for clinical decision-making. Based on our model, we infer that the medial tibiofemoral joint degeneration of KOA after PMM may be related to different joint motion angles. Hence, the biomechanical characteristics of the knee joint under different flexion and extension angles and different loads need further exploration for better rehabilitation after knee joint injury.

The limitations of this study should be addressed: this model is a single case study, and the finite element model of the same individual before and after surgery has not been constructed; the knee joint stresses of different injury types have not been compared and analyzed, and the stresses in complex sports have not been investigated.

## Data Availability

The datasets generated and/or analyzed during the current study are available from the corresponding author by reasonable request.
